# Improvement of health literacy and intervention measurements among low socio-economic status women: findings from the MyBFF@home study

**DOI:** 10.1186/s12905-018-0596-y

**Published:** 2018-07-19

**Authors:** Siew Man Cheong, Noor Safiza Mohamad Nor, Mohamad Hasnan Ahmad, Mala Manickam, Rashidah Ambak, Siti Nurbaya Shahrir, Tahir Aris

**Affiliations:** 1 Institute for Public Health, National Institutes of Health, Ministry of Health Malaysia, Centre for Nutrition Epidemiology Research, Jalan Bangsar, 50590 Kuala Lumpur, Malaysia; 2 Faculty of Medicine and Defence Health, National Defence University of Malaysia, Kem Sungai Besi, Kuala Lumpur, Malaysia; 3 Department of Community Health, Universiti Kebangsaan Malaysia Medical Centre, Kuala Lumpur, Malaysia

**Keywords:** Health literacy, Weight loss behavior, Women’s health, Dietary intake behavior, Body composition

## Abstract

**Background:**

Health literacy (HL) consists of different components and associates with several health outcomes, including obesity. It is linked to an individual’s knowledge, motivation, competencies, behavior, and application to everyday life. The present study aimed to determine the change of HL scores and to investigate the difference of intervention outcomes at the weight loss (WL) intervention and WL maintenance phase between the HL groups.

**Methods:**

A total of 322 participants from the MyBFF@home study completed the Newest Vital Sign (NVS) test at baseline. However, only data from 209 participants who completed the NVS test from baseline to WL intervention were used to determine the HL groups. Change of the NVS scores from baseline to WL intervention phase was categorized into two groups: those with HL improvement (increased 0.1 score and above) and those without HL improvement (no change or decreased 0.1 score and more). Independent variables in this study were change of energy intake, nutrient intake, physical activity, anthropometry measurements, and body composition measurements between baseline and WL intervention as well as between WL intervention and WL maintenance. An Independent sample t-test was used in the statistical analysis.

**Results:**

In general, both intervention and control participants have low HL. The study revealed that the intervention group increased the NVS mean score from baseline (1.19 scores) to the end of the WL maintenance phase (1.51 scores) compared to the control group. There was no significant difference in sociodemographic characteristics between the group with HL improvement and the group without HL improvement at baseline. Most of the dietary intake measurements at WL intervention were significantly different between the two HL groups among intervention participants. Physical activity and body composition did not differ significantly between the two HL groups among both intervention and control groups.

**Conclusion:**

There was an improvement of HL during the WL intervention and WL maintenance phase in intervention participants compared to control participants. HL shows positive impacts on dietary intake behavior among intervention participants. New research is suggested to explore the relationship between HL and weight loss behaviors in future obesity intervention studies.

## Background

Health literacy (HL) is defined as “the degree to which an individual has the ability to obtain, communicate, process, and understand basic health information and services to make appropriate health decisions” [1]. Previous studies found that HL was associated with health status and health behaviour among adult population with chronic diseases such as obesity, cardiovascular disease, and diabetes [2–4]. In Malaysia, the rates of being obese and overweight increased from 14.0% and 29.1% to 17.7% and 30.0%, respectively, between 2006 and 2015 [5, 6]. In addition, the rate of obesity among Malaysian women were alarming in 2015 (20.6%) compared to Malaysian men (15.0%) [5, 6]. In 2015, the National Health and Morbidity Survey (NHMS) Malaysia reported the overall prevalence of adequate HL among adults aged 18 years and above was 6.6%, while a higher prevalence of HL among the urban population of Malaysia and the age group of 20-34 years old [6].

Previous studies revealed that HL was associated with obesity in different age groups. School-going adolescents with lower HL score has been associated with an increased risk of being obese compared to those with higher HL score in Taiwan [7]. Another study among native Hawaiians also reported that there was an inverse relationship between HL scores and BMI [8]. HL among parents also affect nutritional status of children. A previous German population representative study found that lower numeracy which is part of HL among parents was significantly associated with having child who either being underweight or overweight due to poorer portion-size estimation and inferior comprehensive of growth chart [9]. However, only several studies on HL were conducted in Malaysia.

Inadequate HL is a key barrier in weight management because successful weight loss intervention requires a high level of compliance to dietary and physical activity recommendations. HL is linked to the ability of individuals to understand and apply health information to practice for disease prevention and health promotion [10]. Past studies have reported the role of HL in dietary behaviours in the United States as well as in several Asian countries (China, Taiwan, Japan, South Korea, and Singapore) [2, 11–13]. Identifying the relationship between HL level and effectiveness of the weight loss intervention was the concern of our study. There is a lack of study about the effect of HL in weight loss behavioural change in intervention, especially in Malaysia except for one on-going intervention among young couples [14]. There are different tools to measure HL of individuals. The most common tools are Test of Functional Health Literacy in Adults (TOFHLA) and Rapid Estimate of Adult Literacy in Medicine (REALM). The Newest Vital Sign (NVS) was used to assess HL among participants in this study because NVS is an easy tool and rapid to identify individuals at risk of low HL. Moreover, it has been validated and pre-tested in several Asian countries such as Singapore and Japan [15, 16]. The present study aimed to determine the change of NVS scores among overweight and obese women, as well as to investigate the difference of intervention outcomes (energy intake, nutrient intake, physical activity, anthropometry and body composition measurements) during the weight loss (WL) intervention and the weight loss (WL) maintenance phase between the HL groups.

## Methods

My Body is Fat and Fabulous at Home (MyBFF@home) is a 12-month community-based obesity intervention study among overweight or obese housewives who lived in the low-cost flats in Kuala Lumpur [17]. Overweight or obese housewives aged 18-59 years old who were living in the selected 16 low-cost flats were invited to participate into the screening of this intervention. The eligible participants were recruited into MyBFF@home with informed consents. The intervention package included individual counselling, self-monitoring tools, and group exercise sessions were conducted to the intervention group. The intervention was implemented for six months and another six months of weight loss sustainability monitoring. The details of the methodology of the MyBFF@home study were reported elsewhere [16]. A total of 328 participants were recruited into MyBFF@home intervention for both intervention and control groups at baseline. Data from 322 participants from the intervention and control groups who completed the HL test at baseline were used for descriptive data analysis. Data of 209 participants who completed the HL tests at baseline and at WL intervention were also used to determine the HL groups (the with HL improvement group and the without HL improvement group).

HL of participants was assessed using the NVS Malay version by scores. Mean NVS scores and HL levels of participants in intervention and control groups were analysed at baseline, at WL intervention, and WL maintenance phases. The independent variables included in the statistical analysis were change of energy intake, nutrient intake, physical activity, anthropometry and body composition measurements between baseline and WL intervention as well as between WL intervention and WL maintenance.

### Health literacy (HL) assessment

HL was assessed using NVS tool. The tool is based on a nutrition label of a standard ice cream nutrition label [18]. Prior to the baseline data collection, the English version NVS questionnaire and nutrition label were translated into the Malay version using back-forward translation. The translated NVS questionnaire was then modified based on the Malay version NVS from the previous study in 2012, which was conducted among adults in the rural area in Malaysia [19]. Then the Malay version nutrition label was also modified to make it concordant with current Malaysian food labelling. The new Malay version of the NVS questionnaire and the nutrition label were pre-tested among 28 overweight or obese women from a similar socio-demographic background for ease of understanding and acceptability through one-on-one interviews. Further modification of NVS questions and nutrition label were made in response to the suggestions or feedback from the pre-test respondents. The research team members also modified the length and clarity of the NVS questions.

The reliability for the Malay NVS was then evaluated using Cronbach’s alpha coefficient among 51 overweight or obese women during the test-retesting stage. The total NVS mean scores for the test and retest were 1.10 ± 1.25 and 1.35 ± 1.57, which indicated no significant differences between the test and retest (t= -1.17, p = 0.249). The results showed an adequate scale of reliability with Cronbach’s alpha coefficient of 0.75 for all six questions (Cronbach’s alpha ≥ 0.70 was considered evidence of adequate reliability [20]). Repeatability of the questionnaire was also assessed. Intraclass Correlation Coefficient (ICC) within three months apart among the same respondents was used as the indicator of reliability. The ICC revealed moderate reliability (0.57, 95% CI: 0.27-0.75) between the test and the retest scores (0.6 to < 0.7 was considered as acceptable reliability and 0.7 to < 0.9 as good reliability).

The NVS score was calculated based on six questions. Each score was given for each correct answer with maximum of six scores [18]. Then the scores were classified based on three cut-off points, 0-1 was equal to limited HL, 2-3 was equal to possible limited HL, and 4-6 was equal to adequate HL. NVS tests were conducted at three time points: at baseline, at WL intervention, and at maintenance phase to assess the change of HL among participants. Change of the NVS scores from baseline to WL intervention phase was categorized into two groups: **HL improvement** (increased 0.1 score and above) and **without HL improvement** (no change or decreased 0.1 score and more).

### Energy and nutrient intake assessment

The assessment of dietary intake was conducted using a 3-day food diary during two weekdays and one weekend. Detailed information of types of food and beverages (quantity consumed and places of having the meal) were obtained by self-reporting. Energy and nutrient intakes were analysed using Nutritionist Pro software version 2.4.

### Physical activity assessment

The assessment of physical activity with different intensity (vigorous, moderate or walking) was conducted using the short version of Malay International Physical Activity Questionnaire. The physical activity level and intensity was calculated in metabolic equivalent task (MET) in minutes based on seven questions. MET values were calculated using the standard formula [21]. Total duration spent (duration in minutes multiply by frequency in days) on vigorous, moderate-intensity activity, as well as walking in the past seven days were multiplied by 8.0, 4.0, and 3.3, respectively. Total physical activity can be computed as the sum of all MET values.

### Anthropometry and body composition measurements

Body weight and body height of the participants were measured using a digital scale (Tanita HD319, Japan) in kilogram (kg) and a SECA Bodymeter in centimetre (cm). Waist circumference was measured using a SECA measuring tape (SECA, Germany) in centimetre (cm). All measurements were assessed twice, and the mean values of the measurements were used in the analysis. The body mass index (BMI) was calculated by dividing body weight in kg by square of body height in meters. BMI was categorised according to the WHO 2009 classification [22]. Body fat percentage, and visceral fat were measured using bioelectrical impedance analyser (In-body 720).

### Statistical Analysis

Data analysis was performed using SPSS version 20.0 for Windows (SPSS Inc., Chicago, IL, USA). Descriptive analysis was performed to determine the mean score of NVS at the three time points for both intervention and control participants. Chi-square and ANOVA analysis were conducted to examine the differences of baseline characteristics between the participants of the HL improvement and the without HL improvement groups. An independent sample t test was used to determine the difference between the HL groups and the change of obesity intervention measurements among participants at WL intervention phase (month-6) and WL maintenance phase (month-12).

## Results

At baseline, more than 70% of the participants from the intervention and control groups have limited HL, about 20% of them have possible limited HL, and about 10% of them have adequate HL. Among the intervention participants, 39.0% of them (i.e. those who completed the NVS test from baseline until WL intervention) were categorised into the group with HL improvement. 36.3% of control participants were also categorised into the group with HL improvement. Table 1 shows that there was no significant difference in sociodemographic characteristics between the two HL groups among participants.

**Table 1:** Socio-demographic characteristics between health literacy groups among participants at baseline (n=209)

	Intervention			Control		
Socio-demographic characteristics	With HL improvement n (%)	Without HL improvement n (%)	p value	With HL improvement n (%)	Without HL improvement n (%)	p value
Age ± SD	41.2 ± 8.1	44.0 ± 7.5	0.060	42.6 ± 8.0	43.5 ± 7.6	0.570
Education						
Primary	5 (4.2)	11 (9.3)	0.499	3	6	0.597
Secondary/ Tertiary	41 (34.8)	61 (51.7)		29	52	
Monthly household						
Income (RM)	1673 ± 633	1951 ± 883	0.067	2503 ± 1745	2109 ± 856	0.156
Marital status						
Married/ Ever Married	45 (38.1)	70 (59.3)	0.841	32 (35.2)	58 (63.7)	-
Single	1 (0.9)	2 (1.7)		1 (1.1)	0	
Number children in the household						
≤ 2	8 (6.8)	15 (12.7)	0.292	8 (9.0)	7(7.9)	0.285
3-4	21 (17.8)	38 (32.2)		16 (18.0)	31 (34.8)	
≥ 5	17 (14.4)	19 (16.1)		8 (9.0)	19 (21.3)	

**Fig. 1:**
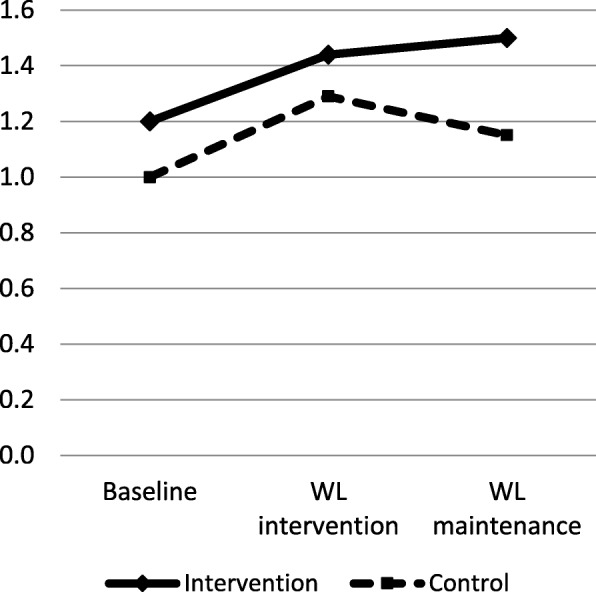
Mean NVS scores at baseline, WL intervention, and WL maintenance among participants from intervention and control groups

Overall, the intervention and control participants had low HL at baseline (below two scores). Figure 1 shows that the intervention group has increased NVS mean scores from the baseline (1.2 scores) to the WL maintenance phase (1.5 scores). However, the control group has fluctuation in the NVS mean scores between the baseline (1.0 scores), the WL intervention phase (1.3 scores), and the WL maintenance phase (1.2 scores).

Table 2 shows that dietary intake measurements at WL intervention phase were significantly different between two HL groups except for cholesterol, vitamin C, and iron intake in the intervention group. However, only carbohydrate intake at WL maintenance phase was significantly different between the two HL groups in the control group.

**Table 2:** Difference between dietary intake and health literacy groups at WL intervention and WL maintenance phase among participants (n=209)

	Intervention					Control				
Intervention measurements	Mean change ± SD					Mean change ± SD				
	With HL improvement		Without HL improvement		p value	With HL improvement		Without HL improvement		p value
Calories intake (kcal)										
WL intervention	459	±508	156	±445	0.004*	133	±434	166	±477	0.755
WL maintenance	36	±354	-12	±312	0.550	238	±492	38	±319	0.145
Protein intake (g)										
WL intervention	15.4	±21.8	5.7	±20.8	0.040*	4.3	±16.0	7.1	±21.6	0.537
WL maintenance	0	±16.2	2.0	±13.8	0.587	3.1	±20.5	-0.5	±18.1	0.450
Carbohydrate intake (g)										
WL intervention	56.9	±67.1	13.5	±73.4	0.007*	11.5	±75.7	16.4	±73.2	0.773
WL maintenance	8.1	±54.4	-5.2	±47.0	0.273	49.1	±78.0	4.3	±50.5	0.005
Total fat intake (g)										
WL intervention	17.2	±23.8	7.0	±19.5	0.030*	6.7	±18.1	5.4	±19.1	0.754
WL maintenance	1.2	±14.6	1.4	±13.7	0.946	3.2	±19.1	4.1	±16.2	0.843
Dietary fiber intake (g)										
WL intervention	2.2	±5.1	0.3	±3.5	0.041*	0.4	±3.6	0.6	±3.7	0.742
WL maintenance	-0.16	±2.4	-1.2	±3.5	0.193	1.5	±3.9	0	±2.6	0.064
Cholesterol intake (mg)										
WL intervention	56.0	±202	23.9	±112.7	0.415	16.3	±148.7	17.0	±167.3	0.983
WL maintenance	4.0	±141.2	3.7	±83.5	0.990	-13.9	±130.4	0.9	±126.7	0.647
Sodium intake (mg)										
WL intervention	444.0	±974.0	27.5	±794.5	0.029*	-66.3	±1077.4	90.5	±936.3	0.492
WL maintenance	-85.1	±665.2	-4.3	±690.0	0.627	293.6	±784.5	-18.0	±636.6	0.077
Vitamin C intake (mg)										
WL intervention	15.4	±59.0	5.9	±81.8	0.568	3.0	±71.9	24.0	±70.3	0.201
WL maintenance	-20.2	±64.3	-24.0	±77.2	0.833	31.5	±72.3	-2.4	±54.7	0.205
Calcium intake (mg)										
WL intervention	115.1	±242.3	-3.9	±230.8	0.023*	38.5	±214.6	102.6	±336.0	0.350
WL maintenance	37.6	±194.0	21.3	±186.5	0.723	54.0	±162.2	11.5	±221.0	0.404
Iron intake (mg)										
WL intervention	4.8	±7.6	2.6	±6.3	0.138	3.0	±6.5	1.9	±6.6	0.477
WL maintenance	-0.7	±4.0	-0.5	±3.4	0.166	0.1	±5.0	-0.4	±5.1	0.689

According to the results, there was no significant difference in physical activity between the two HL groups for both intervention and control groups (p= 0.072-0.515 for intervention group and p=0.883-0.931 for control group). The change of body weight and body composition measurements were similar between the two HL groups among intervention and control participants. There was also no significant difference in anthropometry or body composition measurements between the two HL groups participants (p= 0.100-0.985 for intervention group and p=0.127-0.978 for control group) (Table 3).

**Table 3:** Difference between body composition measurements and health literacy groups at WL intervention and WL maintenance phase among participants (n=209)

	Intervention					Control				
Intervention measurements	Mean change ± SD					Mean change ± SD				
	With HL improvement		Without HL improvement		p value	With HL improvement		Without HL improvement		p value
Body weight (kg)										
WL intervention	1.20	±2.4	1.10	±2.3	0.790	1.2	±2.6	0.7	±2.6	0.443
WL maintenance	-0.29	±3.1	-0.90	±2.0	0.290	-0.6	±3.7	-0.4	±2.4	0.736
BMI (kg/m^2^)										
WL intervention	0.53	±1.0	0.47	±1.0	0.760	0.5	±1.1	0.3	±1.2	0.466
WL maintenance	-0.11	±1.3	-0.38	±0.8	0.246	-0.3	±1.6	-0.2	±1.0	0.712
Waist circumference (cm)										
WL intervention	3.13	±4.95	3.48	±5.2	0.710	2.9	±5.7	2.8	±6.4	0.978
WL maintenance	0.68	±4.90	0.66	±4.7	0.980	0.1	±5.0	1.9	±4.8	0.127
Body fat %										
WL intervention	1.12	±2.8	1.90	±4.4	0.350	0.5	±1.5	0.9	±1.8	0.461
WL maintenance	-0.78	±3.7	-0.30	±4.2	0.638	-0.7	±1.5	-0.1	±2.3	0.223
Visceral fat (cm^2^)										
WL intervention	4.89	±7.2	4.87	±5.8	0.985	5.2	±11.7	3.9	±6.6	0.559
WL maintenance	-0.46	±5.2	-3.09	±6.7	0.100	-2.8	±8.9	-0.7	±6.2	0.308

## Discussion

This study found that more than 70% of intervention and control participants have low HL (below two scores) at baseline, and only about 10% of them have adequate HL. The findings from this study are comparable with a previous national study in Malaysia [6]. However, the prevalence of adequate HL in the study is still lower than other countries, including Singapore (76.4%) [23], Turkey, (28.1%) [24], and Brazil (48.7%) [25].

Overall, the MyBFF@home obesity intervention package improved the HL level of the intervention participants from the baseline to the WL maintenance phase compared to the control participants. The possible reason may be due to our intervention package being designed to be more comprehensive to participants with low HL. The information and advice on healthy living as found in the leaflets and booklets provided by the research team members may have helped the participants understand how to cultivate basic healthy lifestyle habits. Intervention participants may have also improved their HL through participation in the individual diet and physical activity counselling sessions, which included guidance on interpreting food labels, calculating portion size, calculating steps and time of physical activity, and self-monitoring of body weight [17] during WL intervention.

When we look in depth into the change of NVS scores among participants, HL have significant impact on dietary behaviour among intervention participants. This study revealed that the intervention participants with the HL improvement tended to improve intake of most nutrients at the WL intervention phase. This study was comparable with a previous study that discovered that HL has a positive association with diet quality in Delta [26]. In addition, a systematic review revealed that there was a positive, but weak, association between dietary behaviour and nutrition knowledge, which is one of the terms under the umbrella of health literacy [27]. Better nutrition knowledge was often associated with a higher intake of fruits and vegetables [27]. Furthermore, a recent review article found that HL has a positive impact on dietary adherence and other nutrition behaviours only among the general population but not among patients [28]. This review article also found that nutrition intervention among people with diseases is less likely to be affected based on HL [28]. The findings from this review study would explain why nutrient intake among MyBFF@home intervention participants differed significantly between the two HL groups.

According to the findings of this study, the change in physical activity was similar between the two HL groups among both intervention and control participants. In addition, there was a lack of sustainability in physical activity compared to dietary intake behaviour among the intervention participants. A previous national survey in the United States found that adults with higher BMI have higher odds ratios of self-reported low back pain [29]. A possible explanation could be that many of the participants already suffer from body pain, most commonly knee pain, prior to the intervention. The body pain may pose a physical and psychological barrier for the participants to adopt regular exercise habits. Another reason for our findings may be that overweight or obese participants experienced more body pain after increasing physical activity. Therefore, although the intervention participants were adequately trained in basic workouts (dumb bell exercise and walking), the subsequent body pain experienced may become a barrier in developing adherence to regular physical activity.

This study also demonstrated that change in body composition measurements were similar between the two HL groups among both intervention and control participants. They lost body weight and fat during WL intervention and regained body weight and fat during WL maintenance phase. These findings were consistent with a previous weight management intervention among women in the United Kingdom [11]. This may be due to participants only adapting to certain weight loss behaviour but failing to comply holistically with other components of the weight loss programme. In addition, review studies found that weight loss behavioural change and weight loss maintenance are linked to psychological factors [30, 31]. Merely telling people how to lead a healthy lifestyle and teaching them skills in adapting recommended weight loss behaviour is not sufficient to maintain weight loss. Self-efficacy [32] and self-motivation is needed to transform knowledge into long-term action and thereby cause successful and long-lasting behaviour change in intervention programmes [30]. Participants from our study may lack the self-motivation and internal satisfaction to sustain weight loss behaviour during WL maintenance phase. Long-term sustainability of weight loss behaviour may need to be emphasized in future research.

Although there were several previous studies in Malaysia investigating the association between HL, demographic factors and health-related behaviours from adolescents to the elderly population, there is no local study to explore the influence of HL in weight loss behaviour [12, 33, 34]. To our knowledge, this is the first study in Malaysia that investigates the impact of HL in community-based obesity intervention for three time points, from baseline to WL maintenance phase at 12-months. In addition, this study found that the intervention activities that involved complying to portion size control, calculating physical activity durations, monitoring body weight, and reading nutrition labels, which required numeracy skill and comprehension of new health information, improved the HL status of the intervention participants. The findings of this study could be useful to the current obesity prevention or intervention programmes in enhancing adherence of weight loss behaviour among participants.

Our study has several limitations. Although NVS was used to assess HL, there is no gold standard in HL assessment. Different HL assessment tools have different scoring and cut-offs. Another limitation is that it is not known how other target groups (for instance men) may respond in terms of HL status and behavioural change. We only measured the HL improvement from baseline to the end of WL intervention but we did not investigate whether HL improvement in the maintenance phase plays a role in the sustainability of weight loss behaviour because it is not the objective of this study. Future research is warranted to investigate the impact of HL on weight loss behaviour adherence including dietary intake behaviour and physical activity to improve the effectiveness of the obesity intervention.

## Conclusion

There was improvement in HL at the end of intervention and maintenance phase among intervention participants. Overall, HL only influenced dietary intake behaviours among intervention participants compared to control participants. The current study emphasised the need for further research focusing on the role of HL status in obesity intervention and further explored the relationship between HL and physical activity in obesity intervention. Understanding this relationship can help to improve the development of appropriate and effective intervention for overweight or obese participants.

## Abbreviations

MyBFF@home: My Body is Fat and Fabulous at Home
HL: Health literacy
BMI: Body mass index
WL: Weight loss
NHMS: National Health and Morbidity
TOFHLA: Test of Functional Health Literacy in Adults
REALM: Rapid Estimate of Adults Literacy in Medicine
NVS: Newst Vital Sign
MET: metabolic equivalent task
ICC: intraclass correlation coefficient
